# *GNPDA2* Gene Affects Adipogenesis and Alters the Transcriptome Profile of Human Adipose-Derived Mesenchymal Stem Cells

**DOI:** 10.1155/2019/9145452

**Published:** 2019-08-01

**Authors:** Lijun Wu, Feifei Ma, Xiaoyuan Zhao, Mei-Xian Zhang, Jianxin Wu, Jie Mi

**Affiliations:** ^1^Department of Epidemiology, Capital Institute of Pediatrics, Beijing, China; ^2^Department of Biochemistry and Immunology, Capital Institute of Pediatrics, Beijing, China

## Abstract

**Background:**

Genome-wide association studies have found an obesity-related single-nucleotide polymorphism rs10938397 near the glucosamine-6-phosphate deaminase 2 gene (*GNPDA2*) encoding, an enzyme that catalyzes the deamination of the glucosamine-6-phosphate involved in the hexosamine signaling pathway, but the molecular mechanisms underlying the missing link between *GNPDA2* and obesity remain elusive.

**Methods:**

As obesity is accompanied by an increase in the size and the number of adipocytes, the present study investigates the possible mechanism of the *GNPDA2* in adipogenesis using GeneChip® Human Transcriptome Array 2.0 in human adipose-derived mesenchymal stem cells.

**Results:**

We found that overexpression of *GNPDA2* enhanced accumulation of lipid droplets, and knocking down the gene decreased accumulation of lipid droplets. GO term enrichment analysis indicated that most differentially expressed genes (DEGs) affected by deficiency of *GNPDA2* have functions to lipid and glucose metabolism. Further KEGG enrichment analysis showed that the greatest proportion of DEGs are involved in thermogenesis, peroxisome proliferator-activated receptor (PPAR) signaling pathway, carbon metabolism, and fatty acid metabolism including fatty acid degradation, elongation, and biosynthesis.

**Conclusion:**

These findings suggest that *GNPDA2* may be a critical gene for lipid and glucose metabolism, and the expression level of *GNPDA2* alters the transcriptome profile of human adipose-derived mesenchymal stem cells.

## 1. Introduction

In recent years, the increasing prevalence of obesity is a major threat to public health, and childhood obesity has reached epidemic proportions globally [1]. Childhood obesity strongly predisposes to adult diseases including obesity, type 2 diabetes, and hypertension [2–4].

Excess adipocyte number or size is a hallmark of obesity, and the mechanism of adipocyte differentiation has been extensively studied by the identification of the factors or pathways related to adipogenesis. Previous studies have shown that peroxisome proliferator-activated receptor-*γ* (PPAR-*γ*) plays a central role in the regulation of adipocyte differentiation and is highly expressed in adipose tissue [5], but the pathology of obesity remains to be further studied.

In past years, multiple single-nucleotide polymorphisms (SNPs) related to obesity have been identified by genome-wide association studies [6–8]. Among those identified SNPs, the SNP rs10938397, located near the glucosamine-6-phosphate deaminase 2 gene (*GNPDA2*), showed a significant association with obesity in Chinese adults and children [9, 10]. The SNP rs10938397 is also associated with an increased risk of pediatric-onset type 2 diabetes in the Mexican population [11]. *GNPDA2* encoding, an enzyme that catalyzes the deamination of the glucosamine-6-phosphate, is located at chromosome 4p12 (NC_000004.12, 44701795..44726634). GNPDA2 is part of the hexosamine signaling pathway, which is one of the main nutrient-sensing pathways in organisms [12]. However, the molecular mechanisms of the expression of *GNPDA2* involved in obesity are not understood.

Given that adipocytes are thought to differentiate from adipose-derived mesenchymal stem cells (ADMSCs) and *GNPDA2* is related to obesity, we constructed *GNPDA2* overexpression and short hairpin RNA (shRNA) knockdown ADMSCs and analyzed the gene expression profiling. The present study attempts to provide a genetic data towards the possible mechanism of the role of *GNPDA2* in adipogenesis.

## 2. Materials and Methods

All procedures were performed according to standard protocols or the manufacturers' instructions.

The study was approved by the ethics committees of the Capital Institute of Pediatrics.

### 2.1. Cell Culture

ADMSCs (Cyagen Biosciences) were cultured in medium containing Dulbecco's Modified Eagle Medium (DMEM), 10% foetal bovine serum (FBS), 100 *μ*g/mL penicillin, and 100 *μ*g/mL streptomycin and were incubated at 37°C in humidified air containing 5% CO_2_. Human ADMSC adipogenic differentiation medium (HUXMD-90031) was purchased from Cyagen Biosciences, Beijing, China. To stimulate differentiation to adipocytes, cells were induced by medium A containing insulin, dexamethasone, xanthine, glutamine, and rosiglitazone in basal medium. After 3 days, this medium was changed to medium B containing insulin and glutamine in basal medium. Medium B was renewed every 2 days. Differentiation of adipocytes was detected via Oil Red O staining and was viewed with a phase contrast microscope.

### 2.2. Transfection of ADMSCs with Lentiviral Vectors

#### 2.2.1. *GNPDA2* Overexpression

Recombinant lentivirus vector (pLV[Exp]-EGFP:T2A:Puro) containing the coding sequence of human *GNPDA2* longest transcript (NM_138335.2, https://www.ncbi.nlm.nih.gov/gene/?term=NM_138335.2) under the control of EF1A promoter was generated (Cyagen Biosciences, Guangzhou, China). A noncoding vector was used to produce control vector. The *GNPDA2* overexpression vector and the noncoding vector were packaged into third generation lentivirus particles.

ADMSCs were seeded into a 12-well plate (2 × 104 cells per well) and cultured in 1 mL of complete medium at 37°C with 5% CO_2_ overnight.

When inducing differentiation to adipocytes, ADMSCs were transfected with either pLV[Exp]-EGFP:T2A:Puro-EF1A > hGNPDA2 to overexpress *GNPDA2* (OEG) or pLV[Exp]-EGFP:T2A:Puro-Null as a vector control (V1). The optimal virus titer used for cell transfection was screened according to the manufacturer's instructions. After ADMSCs were transfected with lentivirus for 24 hours, the medium was replaced with fresh complete medium. The green fluorescent protein (GFP) expression was visualized using a fluorescent microscope at 72 hours. Triplicate cell cultures were infected with lentiviruses at equal titers using MOI of 2.0. Vector expression was confirmed by both quantitative real-time PCR (RT-PCR) and western blot analysis.

#### 2.2.2. *GNPDA2* shRNA Knockdown

Commercially available lentiviral vectors expressing shRNAs against *GNPDA2* under the control of the U6 promoter were engineered containing GFP as a reporter (Cyagen Biosciences, Guangzhou, China). The shRNA sequence was designed, as follows: ACGGGAATGCTGCAGATTTACCTCGAGGTAAATCTGCAGCATTCCCGT.

The *GNPDA2* knockdown vector and the control vector were packaged into third generation lentivirus particles. ADMSCs were seeded into a 12-well plate (2 × 104 cells per well) and transfected with either pLV[shRNA]-EGFP:T2A:Puro-U6>h*GNPDA2* to knockdown *GNPDA2* (InG) or pLV[shRNA]-EGFP:T2A:Puro-U6>Scramble_shRNA as a vector control (V2) when cells induced differentiation to adipocytes. The optimal virus titer used for cell transfection was screened according to the manufacturer's instructions. Triplicate cell cultures were infected with lentiviruses at equal titers using MOI of 2.0. Vector expression was confirmed by both quantitative RT-PCR and western blot analysis.

### 2.3. Gene Expression Profiling Using Microarrays

The total RNA was extracted from transfected ADMSCs after stimulating differentiation to adipocytes for 10 days, with the TRIzol reagent according to the manufacturer's instructions (Invitrogen). To quantify transcript levels, we carried out profiling with GeneChip® Human Transcriptome Array 2.0 (HTA 2.0, Affymetrix), which contains more than six million distinct oligonucleotide probes with 25 bases per probe [13]. The microarray hybridization was performed according to the manufacturer's standard protocols, and the arrays were scanned by the Affymetrix Scanner 3000 (Affymetrix). The raw data of the HTA 2.0 chips underwent quality control examination and were normalized using robust multiarray analysis for the background correction and quantile algorithm by Transcriptome Analysis Console (version 4.0, Affymetrix) as well as differential expression analysis following the manufacturer's manual.

### 2.4. Identification of Differentially Expressed Genes (DEGs)

The gene-level profiles of the samples from the ADMSCs including OEG, V1, InG, and V2 were analyzed for DEGs using the Transcriptome Analysis Console (version 4.0, Affymetrix) following manufacturer's manual. DEGs with statistical significance between groups (OEG compared with V1 and InG compared with V2) were identified using *p* < 0.05. We defined a fold change (FC) cutoff (FC < −2.0 or >2.0) to filter genes expressed at lower or higher levels.

### 2.5. Analysis of Gene Functions and Pathways

Gene ontology (GO) analysis was applied to determine the roles of the DEGs played in the GO terms [14]. Kyoto Encyclopaedia of Genes and Genomes (KEGG) bioinformatics database [15] was applied to determine the distribution of DEGs in representative pathways. GO and KEGG pathway enrichment analysis of DEGs was performed by clusterProfiler *R* package (R 3.5.0). The Benjamini and Hochberg adjustment was applied to *p* value, and an adjusted *p* value of 0.05 was selected as threshold for significant enrichment results.

### 2.6. Quantitative RT-PCR Analysis

The total RNA was extracted according to the description in the part of “Gene Expression Profiling Using Microarrays.” The cDNA synthesis was performed using a Thermo Scientific RevertAid First Strand cDNA Synthesis Kit (Thermo Fisher Scientific). Expression analysis was performed using the UltraSYBR Mixture (Low ROX) (CWBIO) according to manufacturer's protocol. *GAPDH* was used as internal control. The RT-PCR was carried out in QuantStudio 7 Flex Amplification System (Applied Biosystems), and the differences were calculated using delta-delta CT method. RT-PCR was set up at 95°C for 10 min and then 95°C for 15 s and 60°C for 60 s for 40 cycles. The primers used in this study are listed in Supplementary Table 1. All quantitative RT-PCR were performed in triplicate.

### 2.7. *GNPDA2* Protein Expression

Detection of *GNPDA2* protein expression from transfected ADMSCs after stimulating differentiation to adipocytes for 10 days was carried out by western blot. The procedures were performed according to standard protocols or the manufacturers' instructions. Membranes were blotted with *GNPDA2* polyclonal rabbit antibody (1 : 1000 dilution; 17105-1-AP, Proteintech), followed by goat anti-rabbit IgG HRP conjugate (1 : 5000; Bio-Rad Laboratories). Bands were visualized using the ECL Western Blot Kit (CWBIO, Beijing, China).

### 2.8. Oil Red O Staining

After stimulating differentiation to adipocytes for 10 days, ADMSCs were rinsed twice with PBS and fixed with 4% neutral formaldehyde solution for 30 min at room temperature, rinsed twice with PBS, stained with a filtered Oil Red O solution (stock solution: 5 g/L dissolved in isopropanol alcohol; working solution: Oil Red O stock :distilled water = 3 : 2) for 30 min, rinsed with PBS three times, and visualized under a microscope.

### 2.9. Statistical Analyses

All quantitative data were expressed as mean and standard deviation. The relative expression levels of target genes and the cell supernatant concentration of 8 different inflammatory factors and adipocytokines between groups were compared by *t* test. Level of statistical significance was defined as *p* < 0.05. The data were analyzed using SPSS statistical software (version 18.0, SPSS Inc., Chicago, IL, USA).

## 3. Results

### 3.1. *GNPDA2* Overexpression and *GNPDA2* shRNA Knockdown

To construct the *GNPDA2* overexpression vector, cDNAs coding for the human *GNPDA2* gene was cloned into pLEGFP-T2A and was driven by the EF1A promoter. The vector carried a GFP reporter gene that is promoted by a common cytomegalovirus promoter and downstream of the *GNPDA2* to track transgene expression. To knock down *GNPDA2*, the vector expressing shRNAs against *GNPDA2* under the control of the U6 promoter was engineered containing GFP as a reporter.

**Figure 1:**
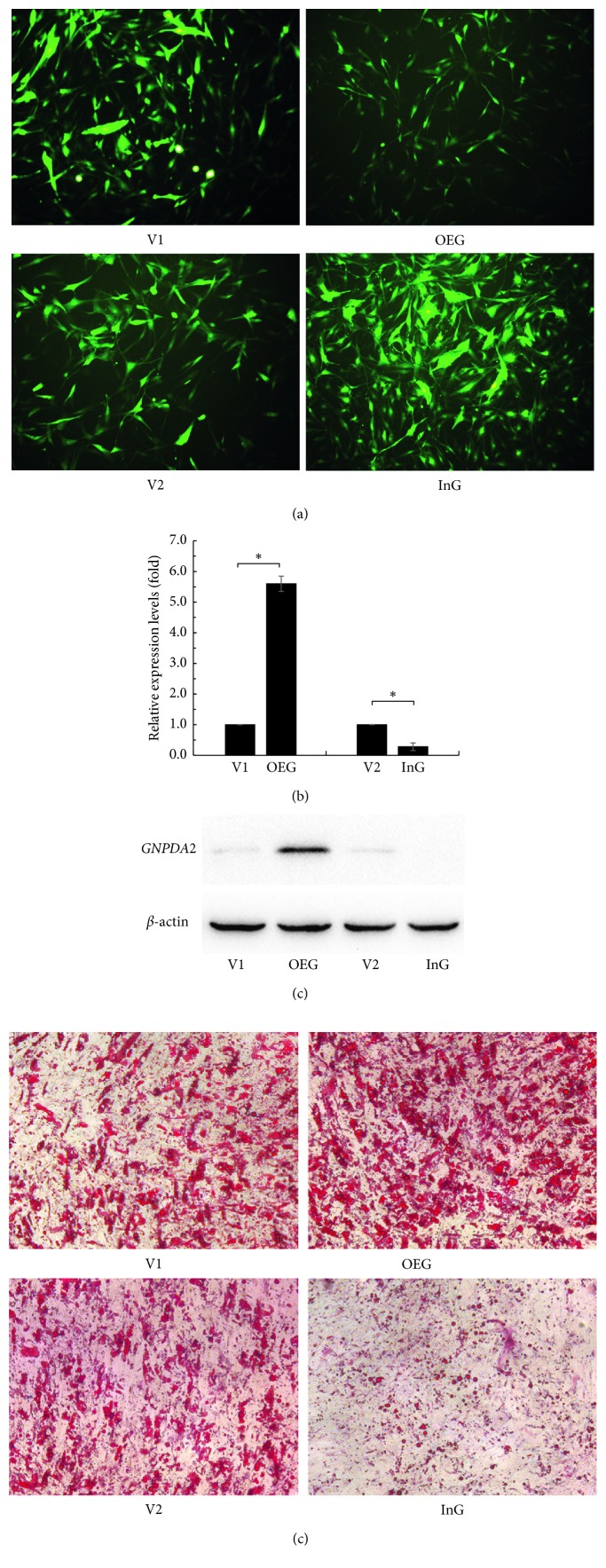
ADMSC transfection and differentiation. (a) GFP expression in OEG, V1, InG, and V2, visualized by fluorescence microscopy. (b) Quantitative RT-PCR analysis of the expression levels of *GNPDA2*. The relative expression is shown as the means ± standard deviation of three independent experiments made in triplicates. ^*∗*^ *p* < 0.05. (c) Western blot analysis of the protein levels of *GNPDA2*. (d) Oil Red O staining after 10 days of culture in adipocyte differentiation inductive medium. OEG: *GNPDA2* overexpressed ADMSCs; V1: the control cells of OEG; InG: *GNPDA2* shRNA knockdown ADMSCs; V2: the control cells of InG.

To verify that *GNPDA2* can be overexpressed or knocked down, the GFP marker was visualized after being transfected with lentivirus for 72 hours (Figure 1(a)). The *GNPDA2* mRNA expression was assessed using quantitative RT-PCR after the transfected ADMSCs were stimulated to differentiate to adipocytes for 10 days (Figure 1(b)). The mRNA expression level of *GNPDA2* in OEG was higher than that in V1 (*p* < 0.05) and that in InG was lower than that in V2 (*p* < 0.05). We also investigated the protein levels of *GNPDA2* in the transfected ADMSCs (Figure 1(c)). Western blot results showed that the exogenous *GNPDA2* protein expression could significantly upregulate the *GNPDA2* protein expression in OEG, and the endogenous *GNPDA2* protein expression was significantly downregulated in InG.

### 3.2. Adipocyte Differentiation of OEG and InG

The adipocyte differentiation was confirmed by Oil Red O staining after 10 days of culture in adipocyte differentiation inductive medium (Figure 1(d)). The differentiation ability of the OEG and InG showed significant difference with the control group V1 and V2, respectively. Enhanced accumulation of lipid droplets was observed after overexpression of *GNPDA2*. Meanwhile, knocking down the gene decreased accumulation of lipid droplets.

To evaluate the biological importance of *GNPDA2* in the modulation of adipogenesis, we used commercial Luminex kits (Cat. # HADCYMAG-61K) to examine cell supernatant concentration of 8 different inflammatory factors and adipocytokines including IL-1*β*, IL-6, leptin, IL-8, adiponectin, resistin, MCP-1, and TNF *α* (Supplementary Table 2). The results indicated that deficiency of *GNPDA2* increased the concentration of IL-1*β*, IL-8, resistin, MCP-1, and TNF *α*, and decreased the concentration of leptin and adiponectin. The overexpression of *GNPDA2* decreased the concentration of IL-1*β*, IL-8, resistin, MCP-1, and TNF-*α* and increased the concentration of leptin and adiponectin. It suggests that inflammatory factors and adipocytokines may mediate the effect of *GNPDA2* on adipogenesis.

In addition, to confirm the adipocyte differentiation, we also measured the expression of marker genes of adipocyte including PPAR-*ɣ* and signal transducer and activator of transcription 5 gene (*STAT5*) (Supplementary Figure 1). The results demonstrated that overexpression of *GNPDA2* upregulated the mRNA expression level of PPAR*-ɣ* and *STAT5*, and deficiency of *GNPDA2* downregulated the mRNA expression level of these genes. The results were consistent with the Oil Red O staining data.

### 3.3. Differentially Expressed Genes

**Figure 2:**
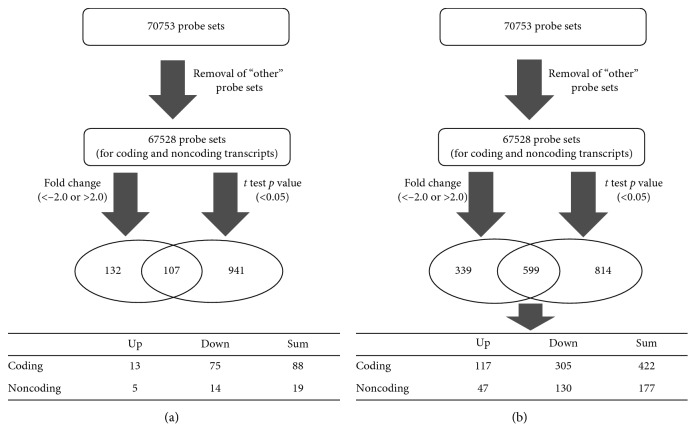
Filtering of probe sets to identify differentially expressed genes in OEG, V1, InG, and V2. We investigated the transcriptome changes with Affymetrix HTA 2.0 microarrays containing 70753 probe sets. If they showed *t* test *p* value <0.05 and a fold change <−2.0 or >2.0, the genes were considered differentially expressed. 107 and 599 probe sets were filtered with these criteria in OEG compared with V1 and InG compared with V2, respectively. The table is giving the numbers of upregulated or downregulated and coding or noncoding transcripts. OEG: *GNPDA2* overexpressed ADMSCs; V1: the control cells of OEG; InG: *GNPDA2* shRNA knockdown ADMSCs; V2: the control cells of InG. (a) OEG vs. V1. (b) InG vs. V2.

Affymetrix microarrays were used to measure the transcriptome in OEG, V1, InG, and V2 cell samples (*n* = 3) in each array sample. Based on the data of the 70753 gene-level probe sets, we searched for genes with significant expression changes in OEG compared with V1 and InG compared with V2, respectively. In total, 107 and 599 probe sets matched the filtering criteria in the two groups (Figure 2), respectively. DEGs with statistical significance between groups (OEG compared with V1 and InG compared with V2) were identified using *p* < 0.05. We defined a FC cutoff (FC < −2.0 or >2.0) to filter genes expressed at lower or higher levels. Supplementary Figure 2 shows the gene expression changes of OEG compared with V1 and InG compared with V2.

To validate the microarray results, 16 DEGs related to lipid or glucose metabolism were selected based on differential expression data. These genes were verified by quantitative RT-PCR in OEG and V1 or InG and V2 cells (Supplementary Figure 3). The results were generally consistent with the microarray data.

### 3.4. Functional Categories of the Genes

To examine what are the DEGs involved in specific biological processes, a GO term enrichment analysis was performed to functional categories and molecular pathways. The DEGs with *p* < 0.05 are listed in Tables 1 and 2, which are categorized by GO analysis of biological processes. As shown in Table 1, overexpression *GNPDA2* is important to multiple biological processes including leukocyte migration, regulation of protein serine/threonine kinase activity, ERK1 and ERK2 cascade, regulation of endocytosis, and regulation of inflammatory response. It suggests that *GNPDA2* may be involved in many cellular events.

In GO analysis, we also found that most DEGs affected by deficiency of *GNPDA2* have functions in fatty acid metabolic process, regulation of lipid metabolic process, lipid modification, lipid localization, fat cell differentiation, regulation of lipid storage, carbohydrate homeostasis, and response to insulin (Table 2). These findings indicate that *GNPDA2* may be a critical gene for lipid and glucose metabolism.

**Figure 3:**
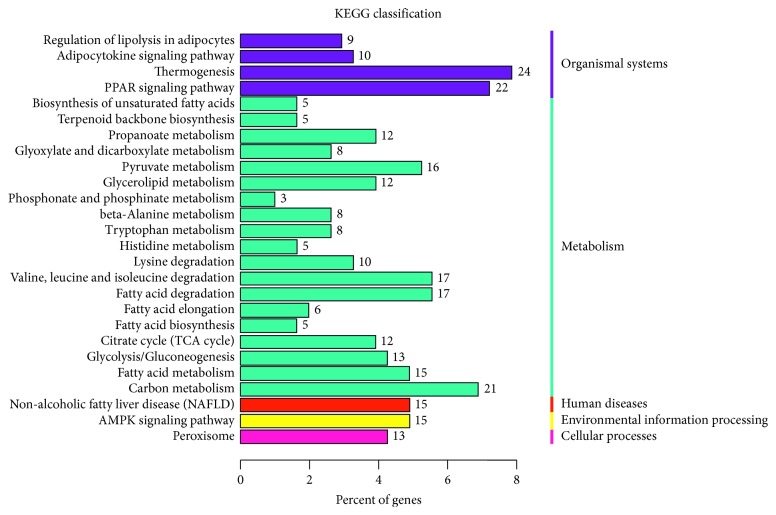
Distribution of differentially expressed genes in representative pathways as compared between InG and V2. KEGG enrichment analysis (http://www.kegg.jp/kegg/kegg1.html) was performed to identify the pathways. InG: *GNPDA2* shRNA knockdown ADMSCs; V2: the control cells of InG.

To identify the distribution of DEGs in representative pathways as compared between InG and V2 cells, the KEGG enrichment analysis was performed. The KEGG pathway analysis is summarized in Figure 3. The greatest proportion of DEGs are involved in thermogenesis, PPAR signaling pathway, carbon metabolism, and fatty acid metabolism including fatty acid degradation, fatty acid elongation, and fatty acid biosynthesis. The DEGs of OEG compared with V1 were also analyzed against the KEGG database for pathway enrichment, but only one pathway was filtered that was unsuitable for this type of KEGG figure.

## 4. Discussion

In this study, we found that overexpression of *GNPDA2* enhanced adipogenesis and knocking down the gene suppressed adipogenesis. Further experiments demonstrated that most DEGs affected by deficiency of *GNPDA2* have functions in fatty acid or lipid metabolism. To our knowledge, this study is the first to investigate the transcriptome changes in *GNPDA2* overexpression and *GNPDA2* shRNA knockdown ADMSCs, and the expression of *GNPDA2* affects the accumulation of lipid droplets and adipogenesis in human adipose-derived mesenchymal stem cells.

According to the results of gene expression profiling, we found that the activated leukocyte cell adhesion molecule gene (ALCAM) was downregulated by overexpression of *GNPDA2* and upregulated by deficiency of *GNPDA2*. ALCAM, a member of the immunoglobulin superfamily, is induced by hypercholesterolemia and is involved in immune responses upon inflammatory stimulation [16]. Currently, ALCAM was identified as a potential mediator in the late complications of diabetes in the kidney [17]. However, there is no sufficient evidence to prove that *GNPDA2* participates in adipogenesis by regulating the expression of ALCAM. The function of *GNPDA2* remains to be further studied to help elucidate the pathogenic role of the gene in obesity.

There are a few limitations to this study. First, the SNP rs10938397 near *GNPDA2* showed a significant association with obesity, but there is no evidence that the SNP rs10938397 may alter *GNPDA2* expression in adipose tissue. Further research should be conducted in future study. Second, DEGs with statistical significance between groups were identified using *p* < 0.05. Benjamini and Hochberg adjustment was not applied to the DEG analysis as it was to the GO/KEGG analysis because only a few genes remained after adjustment. Third, assessment of lipid accumulation is only one marker of adipogenesis, but other supporting data were not provided in our study. The changes in mature adipocyte gene transcripts (including data predifferentiation) should be conducted in further study. Fourth, there were no adipose-specific *GNPDA2* knockout mice data, complementary gene expression data in human adipose tissue from people with and without the risk alleles of rs10938397, and mechanistic data linking *GNPDA2* to altered gene expression profiles in the study. In future studies, the lack of data should be addressed.

## 5. Conclusion

We demonstrate for the first time that the expression of *GNPDA2* affects the accumulation of lipid droplets and adipogenesis in human ADMSCs. 107 and 599 genes were identified to be differentially expressed in the overexpression and deficiency of *GNPDA2* ADMSCs, respectively. The filtered genes comprise genes involved in functional pathways of lipid and glucose metabolism. Our results illustrate that *GNPDA2* may be a critical gene for lipid and glucose metabolism, and the expression level of *GNPDA2* alters the transcriptome profile of human adipose-derived mesenchymal stem cells.

## Figures and Tables

**Table 1:** GO biological processes enrichment analysis results (OEG vs. V1).

ID	Description	Count	*p* value	Differentially expressed genes
GO: 0030100	Regulation of endocytosis	9	6.42*E*−06	SFRP4/GREM1/PRKD1/SERPINE1/ITGA2/PTX3/SGIP1/DKK1/GAS6
GO: 0045807	Positive regulation of endocytosis	7	8.22*E*−06	SFRP4/GREM1/SERPINE1/ITGA2/PTX3/SGIP1/GAS6
GO: 0070371	ERK1 and ERK2 cascade	10	1.11*E*−05	CHI3L1/PTPN22/NEK10/IL6/PDGFD/IGF1/CTGF/HTR2B/MIR222/GAS6
GO: 0051781	Positive regulation of cell division	6	1.23*E*−05	TGFB2/FGF7/PDGFD/HTR2B/FGF5/EREG
GO: 0050900	Leukocyte migration	12	2.99*E*−05	IL6/IGLV3-25/TGFB2/IGHV1-69/GREM1/PDGFD/SERPINE1/ITGA2/B4GALT1/RPS19/SCG2/GAS6
GO: 0051897	Positive regulation of protein kinase B signaling	7	4.03*E*−05	CHI3L1/IL6/FGF7/FGF5/MIR222/EREG/GAS6
GO: 0071900	Regulation of protein serine/threonine kinase activity	11	2.17*E*−04	CHI3L1/PTPN22/NEK10/RGS4/CEMIP/HSPB1/PDGFD/IGF1/HTR2B/FAM20A/DKK1
GO: 0030595	Leukocyte chemotaxis	7	2.45*E*−04	IL6/TGFB2/GREM1/SERPINE1/RPS19/SCG2/GAS6
GO: 0050727	Regulation of inflammatory response	9	5.79*E*−04	IL6/IGLV3-25/SOCS5/IGHV1-69/SERPINE1/ITGA2/RPS19/MIR222/NR1D2
GO: 0032675	Regulation of interleukin-6 production	5	5.89*E*−04	PTPN22/IL6/SOCS5/EREG/GAS6
GO: 0002685	Regulation of leukocyte migration	6	6.29*E*−04	IL6/GREM1/PDGFD/SERPINE1/ITGA2/GAS6
GO: 0032635	Interleukin-6 production	5	8.39*E*−04	PTPN22/IL6/SOCS5/EREG/GAS6
GO: 0006809	Nitric oxide biosynthetic process	4	8.80*E*−04	CYP1B1/IL6/DDAH1/PTX3
GO: 0002687	Positive regulation of leukocyte migration	5	9.38*E*−04	IL6/PDGFD/SERPINE1/ITGA2/GAS6
GO: 1900165	Negative regulation of interleukin-6 secretion	2	1.01*E*−03	PTPN22/GAS6
GO: 0014065	Phosphatidylinositol 3-kinase signaling	5	1.04*E*−03	IER3/TGFB2/PDGFD/IGF1/HTR2B
GO: 0046209	Nitric oxide metabolic process	4	1.08*E*−03	CYP1B1/IL6/DDAH1/PTX3
GO: 0061097	Regulation of protein tyrosine kinase activity	4	1.08*E*−03	SOCS5/GREM1/EREG/GAS6
GO: 2001057	Reactive nitrogen species metabolic process	4	1.26*E*−03	CYP1B1/IL6/DDAH1/PTX3
GO: 0010692	Regulation of alkaline phosphatase activity	2	1.29*E*−03	TGFB2/ITGA2
GO: 0070886	Positive regulation of calcineurin-NFAT signaling cascade	2	1.29*E*−03	LMCD1/IGF1
GO: 0071902	Positive regulation of protein serine/threonine kinase activity	8	1.36*E*−03	CHI3L1/NEK10/CEMIP/PDGFD/IGF1/HTR2B/FAM20A/DKK1
GO: 0002090	Regulation of receptor internalization	3	1.59*E*−03	SFRP4/GREM1/DKK1
GO: 0032715	Negative regulation of interleukin-6 production	3	1.59*E*−03	PTPN22/SOCS5/GAS6
GO: 0060394	Negative regulation of pathway-restricted SMAD protein phosphorylation	2	1.61*E*−03	GREM1/DKK1
GO: 0031952	Regulation of protein autophosphorylation	3	1.99*E*−03	NEK10/GREM1/PDGFD
GO: 0045429	Positive regulation of nitric oxide biosynthetic process	3	2.13*E*−03	IL6/DDAH1/PTX3

The differentially expressed genes with *p* < 0.05 are listed in the table, which are categorized by GO analysis of biological processes. For example, 9 of the filtered genes belonged to the category “regulation of endocytosis.” The 9 genes are proportionally more than in the reference gene set as specified by *p* value (6.42*E*−06).

**Table 2:** GO biological processes enrichment analysis results (InG vs. V2).

ID	Description	Count	*p* value	Differentially expressed genes
GO: 0006631	Fatty acid metabolic process	59	5.73*E*−25	PDK4/ETFA/GHR/HACD2/ABHD5/ABCD2/LEP/LIPE/ECHDC1/ACSL4/ETFDH/CNR1/ACSF2/DECR1/ACAA2/ACACB/HADH/HACD1/ACADM/PPARG/LPL/DLAT/PHYH/IRS2/ACAT2/PRKAR2B/ACSL3/AACS/ALDH3A2/PNPLA3/ADIPOQ/MLXIPL/NDUFAB1/ACOX1/HSD17B4/ACSS2/HACL1/MLYCD/FADS2/ACADSB/PDPN/FABP3/PDHB/PDHX/ACSL1/CPT2/LPIN1/PCCA/ACOT1/SREBF1/DGAT2/ECHS1/ACADL/GPAM/MSMO1/ADIPOR2/DLD/PDHA1/OLAH

GO: 0019395	Fatty acid oxidation	29	4.74*E*−20	PDK4/ETFA/ABCD2/LEP/ECHDC1/ETFDH/CNR1/DECR1/ACAA2/ACACB/HADH/ACADM/PPARG/PHYH/IRS2/ACAT2/ALDH3A2/ADIPOQ/NDUFAB1/ACOX1/HSD17B4/HACL1/MLYCD/FABP3/CPT2/DGAT2/ECHS1/ACADL/ADIPOR2

GO: 0034440	Lipid oxidation	29	8.88*E*−20	PDK4/ETFA/ABCD2/LEP/ECHDC1/ETFDH/CNR1/DECR1/ACAA2/ACACB/HADH/ACADM/PPARG/PHYH/IRS2/ACAT2/ALDH3A2/ADIPOQ/NDUFAB1/ACOX1/HSD17B4/HACL1/MLYCD/FABP3/CPT2/DGAT2/ECHS1/ACADL/ADIPOR2

GO: 0019216	Regulation of lipid metabolic process	45	1.67*E*−14	PDK4/ABHD5/ABCD2/PDE3B/LEP/CNR1/HCAR2/SORBS1/ACACB/ACADM/PPARG/DLAT/ANGPTL4/IRS2/IDH1/PPP2R5A/LYN/NCOA1/NSMAF/ACSL3/LSS/ADIPOQ/MLXIPL/THRSP/ACOX1/IDI1/LGALS12/FDPS/ME1/MLYCD/FABP3/PDHB/PDHX/ACSL1/CPT2/PNPLA2/SREBF1/DGAT2/NR1D1/ACADL/GPAM/ADIPOR2/FDFT1/DLD/PDHA1

GO: 0019217	Regulation of fatty acid metabolic process	19	3.28*E*−11	PDK4/ABCD2/CNR1/ACACB/PPARG/DLAT/IRS2/ADIPOQ/MLXIPL/MLYCD/FABP3/PDHB/PDHX/SREBF1/DGAT2/ACADL/ADIPOR2/DLD/PDHA1

GO: 0030258	Lipid modification	31	2.90*E*−09	PDK4/ETFA/ABCD2/LEP/KLB/ECHDC1/ETFDH/CNR1/DECR1/ACAA2/ACACB/HADH/ACADM/PPARG/PTEN/PHYH/IRS2/ACAT2/ALDH3A2/ADIPOQ/NDUFAB1/ACOX1/HSD17B4/HACL1/MLYCD/FABP3/CPT2/DGAT2/ECHS1/ACADL/ADIPOR2

GO: 0032868	Response to insulin	29	4.08*E*−09	PDK4/UCP2/PCK1/PDE3B/LEP/SOS1/SLC25A33/PFKFB1/ACVR1C/SORBS1/HADH/ATP6V1D/PPARG/PTEN/IRS2/LYN/KANK1/CAT/ADIPOQ/CPEB1/CRY1/KAT2B/FABP3/LPIN1/SREBF1/SORT1/DENND4C/SIK2/OPA1

GO: 0046320	Regulation of fatty acid oxidation	10	1.15*E*−08	PDK4/ABCD2/CNR1/ACACB/PPARG/IRS2/MLYCD/FABP3/DGAT2/ACADL

GO: 0019915	Lipid storage	12	9.09*E*−07	HILPDA/ABHD5/LEP/ACVR1C/ACACB/PPARG/LPL/LDAH/OSBPL11/CRY1/PNPLA2/DGAT2

GO: 0032869	Cellular response to insulin stimulus	21	2.21*E*−06	PDK4/UCP2/PCK1/PDE3B/LEP/SOS1/SLC25A33/SORBS1/ATP6V1D/PPARG/PTEN/IRS2/KANK1/ADIPOQ/CPEB1/KAT2B/LPIN1/SREBF1/DENND4C/SIK2/OPA1

GO: 0045834	Positive regulation of lipid metabolic process	16	3.13*E*−06	ABHD5/ABCD2/SORBS1/PPARG/IRS2/LYN/NSMAF/ACSL3/ADIPOQ/MLXIPL/MLYCD/FABP3/PNPLA2/SREBF1/DGAT2/NR1D1

GO: 0010889	Regulation of sequestering of triglyceride	5	2.59*E*−05	ABHD5/PPARG/LPL/OSBPL11/PNPLA2

GO: 0010876	Lipid localization	29	2.94*E*−05	HILPDA/ABHD5/ABCD2/LEP/ACSL4/ACVR1C/ACACB/PPARG/LPL/LDAH/OSBPL11/IRS2/FZD4/CHKA/NCOA1/ACSL3/ADIPOQ/RBP4/THRSP/CRY1/FABP3/ACSL1/CPT2/ATP11C/PNPLA2/DGAT2/BDKRB2/ABCA10/PITPNA

GO: 0015909	Long-chain fatty acid transport	10	3.94*E*−05	ABCD2/ACACB/PPARG/IRS2/ACSL3/THRSP/FABP3/ACSL1/CPT2/BDKRB2

GO: 0015908	Fatty acid transport	12	4.62*E*−05	ABCD2/LEP/ACSL4/ACACB/PPARG/IRS2/ACSL3/THRSP/FABP3/ACSL1/CPT2/BDKRB2

GO: 0010883	Regulation of lipid storage	8	6.12*E*−05	HILPDA/ABHD5/LEP/ACACB/PPARG/LPL/OSBPL11/PNPLA2

GO: 0045444	Fat cell differentiation	19	7.69*E*−05	ADIRF/FBXO9/ITGA6/LEP/PPARG/FABP4/OSBPL11/MIR29B1/SULT1E1/AACS/ADIPOQ/LGALS12/ARNTL/TMEM120A/SREBF1/NR1D1/LMO3/SORT1/SFRP2

GO: 0030730	Sequestering of triglyceride	5	9.02*E*−05	ABHD5/PPARG/LPL/OSBPL11/PNPLA2

GO: 0010891	Negative regulation of sequestering of triglyceride	3	3.46*E*−04	ABHD5/PPARG/PNPLA2

GO: 0033500	Carbohydrate homeostasis	18	3.88*E*−04	PDK4/OXCT1/UCP2/PCK1/LEP/CNR1/PPARG/EFNA5/IRS2/AACS/ADIPOQ/MLXIPL/RBP4/CRY1/PYGL/NR1D1/ADIPOR2/OPA1

GO: 0042593	Glucose homeostasis	18	3.88*E*−04	PDK4/OXCT1/UCP2/PCK1/LEP/CNR1/PPARG/EFNA5/IRS2/AACS/ADIPOQ/MLXIPL/RBP4/CRY1/PYGL/NR1D1/ADIPOR2/OPA1

GO: 0070542	Response to fatty acid	10	4.14*E*−04	PDK4/UCP2/PPARG/CAT/AACS/ADIPOQ/FABP3/ACSL1/SREBF1/DGAT2

GO: 0055088	Lipid homeostasis	12	4.75*E*−04	PPARG/LPL/FABP4/ANGPTL4/IRS2/PNPLA3/MLXIPL/FABP3/PNPLA2/DGAT2/NR1D1/GPAM

GO: 0045923	Positive regulation of fatty acid metabolic process	6	5.66*E*−04	ABCD2/PPARG/IRS2/ADIPOQ/MLXIPL/MLYCD

GO: 0045598	Regulation of fat cell differentiation	12	5.99*E*−04	ADIRF/LEP/PPARG/MIR29B1/SULT1E1/ADIPOQ/LGALS12/ARNTL/NR1D1/LMO3/SORT1/SFRP2

GO: 0046321	Positive regulation of fatty acid oxidation	4	9.22*E*−04	ABCD2/PPARG/IRS2/MLYCD

GO: 0010888	Negative regulation of lipid storage	4	2.03*E*−03	ABHD5/LEP/PPARG/PNPLA2

The differentially expressed genes with *p* < 0.05 are listed in the table, which are categorized by GO analysis of biological processes. For example, 59 of the filtered genes belonged to the category “fatty acid metabolic process.” The 59 genes are proportionally more than in the reference gene set as specified by *p* value (5.73*E*−25).

## Data Availability

The data used to support the findings of this study are available from the corresponding author upon request.

## Supplementary Materials

Supplementary Materials Supplementary Table 1: primers used for quantitative real-time PCR analysis. Supplementary Table 2: the cell supernatant concentration of 8 different inflammatory factors and adipocytokines in OEG, V1, InG, and V2. Supplementary Figure 1: quantitative real-time PCR analysis of the expression levels of the marker genes of adipocyte in OEG, V1, InG, and V2. Supplementary Figure 2: volcano plot of gene expression changes. Supplementary Figure 3: the expression levels of 16 genes were validated by quantitative real-time PCR in OEG, V1, InG, and V2.
