# Supplementary material for: GNPDA2 Gene Affects Adipogenesis and Alters the Transcriptome Profile of Human Adipose-Derived Mesenchymal Stem Cells
Source: Int J Endocrinol. 2019 Aug 1;2019:9145452. doi: 10.1155/2019/9145452 (PMC6701328; doi:10.1155/2019/9145452)
Supplement: Supplementary Materials — Supplementary Table 1: primers used for quantitative real-time PCR analysis. Supplementary Table 2: the cell supernatant concentration of 8 different inflammatory factors and adipocytokines in OEG, V1, InG, and V2. Supplementary Figure 1: quantitative real-time PCR analysis of the expression levels of the marker genes of adipocyte in OEG, V1, InG, and V2. Supplementary Figure 2: volcano plot of gene expression changes. Supplementary Figure 3: the expression levels of 16 genes were validated by quantitative real-time PCR in OEG, V1, InG, and V2. [file 9145452.f1.doc]

**Supplementary tables and figures**

Supplementary Table 1: Primers used for quantitative real-time PCR analysis.

| Gene | Primer sequences (5'to3') | Product size (bp) |
| --- | --- | --- |
| *GAPDH* | F: GGAGTCCACTGGCGTCTT | 144 |
|  | R: AGGCTGTTGTCATACTTCTCAT |  |
| *GNPDA2* | F: AATGGGCAGCCAAATACATCTGT | 198 |
|  | R: TTCTTGGAAGTCCTACATATTCATC |  |
| *CHI3L2* | F: GGCCAAGCTCCTACTACAATG | 181 |
|  | R: GACTCTGTGATGGGTCCAGC |  |
| *SFRP4* | F: CCTGGAACATCACGCGGAT | 182 |
|  | R: CGGCTTGATAGGGTCGTGC |  |
| *HP* | F: CAGCACAGTCCCCGAAAAGAA | 173 |
|  | R: CAGTCGCATACCAGGTGTCC |  |
| *ALCAM* | F: TCCTGCCGTCTGCTCTTCT | 128 |
|  | R: TTCTGAGGTACGTCAAGTCGG |  |
| *CHI3L1* | F: GTGAAGGCGTCTCAAACAGG | 147 |
|  | R: GAAGCGGTCAAGGGCATCT |  |
| *FST* | F: ACGTGTGAGAACGTGGACTG | 151 |
|  | R: CACATTCATTGCGGTAGGTTTTC |  |
| *SERPINE1* | F: ACCGCAACGTGGTTTTCTCA | 109 |
|  | R: TTGAATCCCATAGCTGCTTGAAT |  |
| *CYP1B1* | F: TGAGTGCCGTGTGTTTCGG | 197 |
|  | R: GTTGCTGAAGTTGCGGTTGAG |  |
| *ACACB* | F: GCCTCTGATAACTCAGGGGAG | 215 |
|  | R: CCAGTCCCGTTGGCTTGAA |  |
| *RETSAT* | F: TACTTGGGACTATTCTCTGGCA | 133 |
|  | R: GCACTTGGTTGGCTGAAAAAG |  |
| *PPARG* (PPAR-ɣ) | F: GGGATCAGCTCCGTGGATCT | 186 |
|  | R: TGCACTTTGGTACTCTTGAAGTT |  |
| *PLIN4* | F: GGCACCAAGAACACTGTCTG | 106 |
|  | R: TCGTACCCATGACCATAGACTT |  |
| *THRSP* | F: CAGGTGCTAACCAAGCGTTAC | 108 |
|  | R: CAGAAGGCTGGGGATCATCA |  |
| *MFAP5* | F: GGGTCAATAGTCAACGAGGAGA | 90 |
|  | R: CTGTAGCGGGATCATTCACCA |  |
| *DGAT2* | F: ATTGCTGGCTCATCGCTGT | 131 |
|  | R: GGGAAAGTAGTCTCGAAAGTAGC |  |
| *CIDEC* | F: AAGTCCCTTAGCCTTCTCTACC | 154 |
|  | R: CCTTCCTCACGCTTCGATCC |  |
| *STAT5* | F: AAATGAGAACACCCGCAACGA | 131 |
|  | R: CAGCACGCTTGATCCTCTTCAG |  |

Supplementary Table 2: The cell supernatant concentration of 8 different inflammatory factors and adipocytokines in OEG, V1, InG, and V2.

|  | V1 | OEG | *p* | V2 | InG | *p* |
| --- | --- | --- | --- | --- | --- | --- |
| IL-1β | 0.59 ±0.07 | 0.53±0.10 | 0.342 | 0.62±0.05 | 0.90±0.23 | 0.099 |
| IL-8 | 476.69±98.78 | 364.34±30.85 | 0.104 | 342.19±46.69 | 649.30±98.90 | **0.008** |
| Resistin | 4.74±0.73 | 3.67±1.24 | 0.138 | 5.10±0.31 | 8.34±1.74 | **0.034** |
| MCP-1 | 7228.33±1039.41 | 5063.06±374.41 | **0.011** | 5670.76±251.77 | 9490.97±1219.74 | **0.006** |
| TNF α | 0.58±0.07 | 0.45±0.04 | **0.015** | 0.56±0.05 | 1.11±0.31 | **0.037** |
| Leptin | 2345.27±1436.39 | 2410.26±830.45 | 0.945 | 3738.67±741.44 | 665.13±296.52 | **0.003** |
| Adiponectin | 9947.29±677.80 | 16813.13±13613.25 | 0.345 | 13918.47±11592.77 | 4864.29±1565.66 | 0.251 |
| IL-6 | 2858.35±831.50 | 1274.09±11.31 | **0.015** | 2222.56±854.94 | 1293.47±236.26 | 0.144 |

All quantitative data were expressed as mean (pg/mL) ±standard deviation. OEG: *GNPDA2* overexpression ADMSCs; V1: the control cells of OEG; InG: *GNPDA2* shRNAs knockdown ADMSCs; V2: the control cells of InG; IL: interleukin; MCP-1: monocyte chemotactic protein 1; TNF α: tumor necrosis factor α. Values in bold are statistically significant.


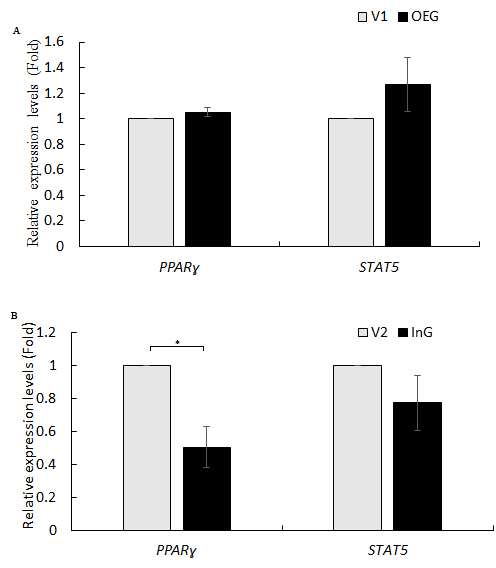


Supplementary Figure 1: Quantitative real-time PCR analysis the expression levels of the marker genes of adipocyte in OEG, V1, InG, and V2. Supplementary figure 1*A* and 1*B* showed the expression levels of the genes of OEG compared with V1 and InG compared with V2, respectively. The relative expression is shown as the means ± standard deviation of three independent experiments made in triplicates. **p*<0.05. PPAR-ɣ: peroxisome proliferator activated receptor ɣ; STAT5: signal transducer and activator of transcription 5. OEG: *GNPDA2* Overexpression ADMSCs; V1: the control cells of OEG; InG: *GNPDA2* shRNAs knockdown ADMSCs; V2: the control cells of InG.


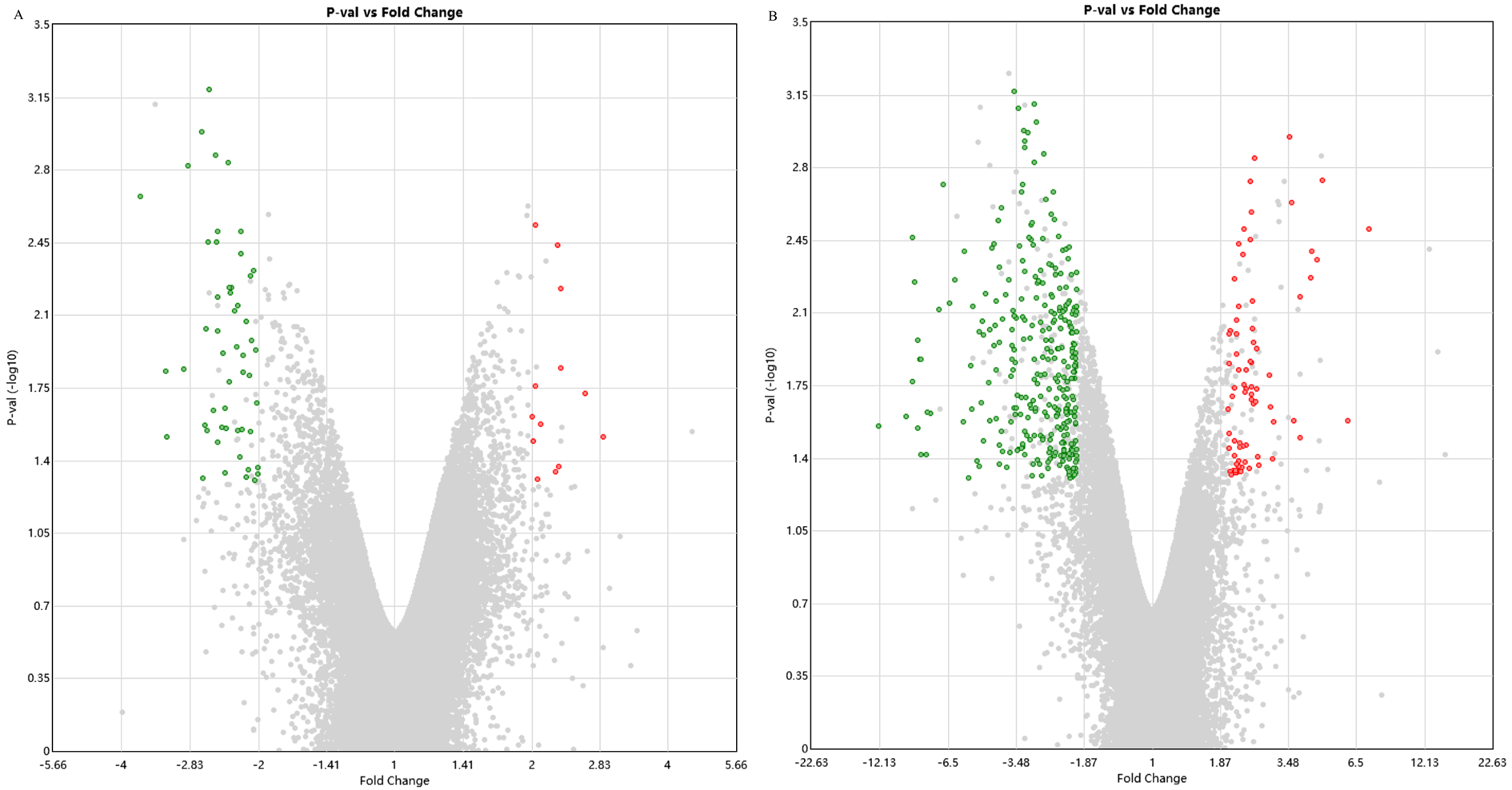


Supplementary Figure 2: Volcano plot of gene expression changes. Figure 2*A* and 2*B* showed the transcriptome of OEG compared with V1 and InG compared with V2, respectively. The x axis specifies the fold changes, and the y axis specifies the negative logarithm to the base 10 of the *t* test *p* values. The red and green dots represent the probe sets for transcripts expressed at significantly higher or lower levels, respectively. The gray dots represent the probe sets that did not pass the filtering criteria. OEG: *GNPDA2* overexpression ADMSCs; V1: the control cells of OEG; InG: *GNPDA2* shRNAs knockdown ADMSCs; V2: the control cells of InG.


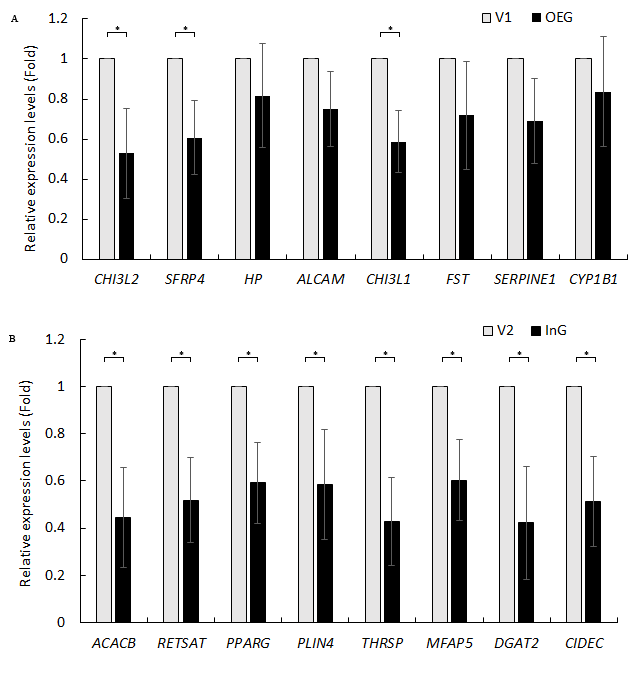


Supplementary Figure 3: The expression levels of 16 genes were validated by quantitative real-time PCR in OEG, V1, InG, and V2. Supplementary figure 3*A* and 3*B* showed the expression levels of 8 genes of OEG compared with V1 and InG compared with V2, respectively. The relative expression is shown as the means ± standard deviation of three independent experiments made in triplicates. **p*<0.05. OEG: *GNPDA2* Overexpression ADMSCs; V1: the control cells of OEG; InG: *GNPDA2* shRNAs knockdown ADMSCs; V2: the control cells of InG.
